# Antimicrobial Protein Candidates from the Thermophilic *Geobacillus* sp. Strain ZGt-1: Production, Proteomics, and Bioinformatics Analysis

**DOI:** 10.3390/ijms17081363

**Published:** 2016-08-19

**Authors:** Rawana N. Alkhalili, Katja Bernfur, Tarek Dishisha, Gashaw Mamo, Jenny Schelin, Björn Canbäck, Cecilia Emanuelsson, Rajni Hatti-Kaul

**Affiliations:** 1 Biotechnology, Department of Chemistry, Lund University, Lund SE-221 00, Sweden; rawana.alkhalili@biotek.lu.se (R.N.A.); tarek.dishisha@pharm.bsu.edu.eg (T.D.); gashaw.mamo@biotek.lu.se (G.M.); 2 Center for Molecular Protein Science, Department of Chemistry, Lund University, Lund SE-221 00, Sweden; katja.bernfur@biochemistry.lu.se (K.B.); cecilia.emanuelsson@biochemistry.lu.se (C.E.); 3 Department of Microbiology and Immunology, Faculty of Pharmacy, Beni-Suef University, Beni-Suef 62511, Egypt; 4 Applied Microbiology, Department of Chemistry, Lund University, Lund SE-221 00, Sweden; jenny.schelin@tmb.lth.se; 5 Department of Biology, Microbial Ecology Group, Lund University, Lund SE-221 00, Sweden; bjorn.canback@biol.lu.se

**Keywords:** thermophile, *Geobacillus*, antimicrobial proteins, SDS-resistant proteins, immobilization, cell-recycling, food spoilage bacteria

## Abstract

A thermophilic bacterial strain, *Geobacillus* sp. ZGt-1, isolated from Zara hot spring in Jordan, was capable of inhibiting the growth of the thermophilic *G. stearothermophilus* and the mesophilic *Bacillus subtilis* and *Salmonella typhimurium* on a solid cultivation medium. Antibacterial activity was not observed when ZGt-1 was cultivated in a liquid medium; however, immobilization of the cells in agar beads that were subjected to sequential batch cultivation in the liquid medium at 60 °C showed increasing antibacterial activity up to 14 cycles. The antibacterial activity was lost on protease treatment of the culture supernatant. Concentration of the protein fraction by ammonium sulphate precipitation followed by denaturing polyacrylamide gel electrophoresis separation and analysis of the gel for antibacterial activity against *G. stearothermophilus* showed a distinct inhibition zone in 15–20 kDa range, suggesting that the active molecule(s) are resistant to denaturation by SDS. Mass spectrometric analysis of the protein bands around the active region resulted in identification of 22 proteins with molecular weight in the range of interest, three of which were new and are here proposed as potential antimicrobial protein candidates by *in silico* analysis of their amino acid sequences. Mass spectrometric analysis also indicated the presence of partial sequences of antimicrobial enzymes, amidase and dd-carboxypeptidase.

## 1. Introduction

Competition for nutrients and space in a given habitat leads organisms to develop their own strategies for survival and growth, one of which is the secretion of antimicrobial substances resulting in either killing or impairing the growth of competing organisms [1]. These antimicrobial substances possess promising clinical and industrial value [2]. Nowadays, the growing problem of multidrug resistance and increasing skepticism about the use of chemical additives in food products have led to an urgent need for finding new and more effective antimicrobial agents [2]. Exploring new ecological niches offers opportunities for isolating novel microorganisms with potent novel antimicrobial compounds [2]. Extremophiles, microorganisms living in extreme conditions of pH, temperature, salt concentration, etc., represent one such source [3]. A number of studies have reported the production of antibacterial peptides or bacteriocins from extremophiles such as alkaliphilic and thermophilic *Bacillus* species [3,4,5,6,7] and also thermophilic *Geobacillus* species such as *G. thermodentrificans* [8] and *G. stearothermophilus* [9].

Thermophiles represent a source of stable proteins, which help the microorganisms to save energy and nutrient resources that would otherwise be dissipated on protein degradation and synthesis [10]. Investigating the potential of thermophiles to antagonize the growth of other thermophiles, particularly the known food-spoiling thermophilic bacteria, paves the way for identifying new stable food biopreservatives. One of the known thermophilic food-spoiling bacteria is *G.* *stearothermophilus*, which creates problems in the dairy industry due to its ability to form biofilms on the process equipment, resulting in spoilage of the final product [11]. *G.* *stearothermophilus* spores cause spoilage of low-acid canned, and ready-made vegetable- and meat-based meals [11]. In a clinical context, the potential of the antimicrobial molecules from thermophiles to inhibit the growth of mesophilic pathogens would also be an interesting finding.

The present study concerns a thermophilic isolate from Zara hot spring in Jordan, identified as a *Geobacillus* species that displayed antibacterial activity against *G.* *stearothermophilus* and some mesophilic bacterial strains including pathogens. A system for cultivation of the organism for the production of antibacterially active proteins was developed, followed by proteomics and bioinformatics analysis of the expressed proteins to identify the potential antimicrobial candidates.

## 2. Results and Discussion

### 2.1. Isolation, Identification, and Characterization of the Isolate

Isolation of thermophilic bacterial strains from Zara hot spring sample was performed at 60 °C and the isolate *Geobacillus* sp. (designated as *Geobacillus* sp. ZGt-1, GenBank accession no. KT02696) was identified based on 16S rRNA gene sequencing (Table S1), which showed 99.9%–100.0% identity to *G. thermoleovorans* and *G. kaustophilus*, respectively. Since the assignment of *G. thermoleovorans* and *G. kaustophilus* into distinct species has been questioned previously [12], we did not affiliate strain ZGt-1 to any of the species, and designated it as *Geobacillus* sp. ZGt-1. Its cells appeared as single rods or in pairs after an overnight cultivation on R2A/Mueller Hinton agar. The sporulating cells of ZGt-1 showed one terminal endospore per cell. The colonies on Mueller Hinton (MH) agar were yellowish, rounded, raised, entire, and shiny, while they were creamy white, rounded, raised, entire, and opaque on R2A agar. Strain 10 was also isolated from the same hot spring and identified as *G. stearothermophilus* (GenBank accession no. KU933578) (Table S1).

### 2.2. Antibacterial Activity of *Geobacillus* sp. ZGt-1

**Figure 1:**
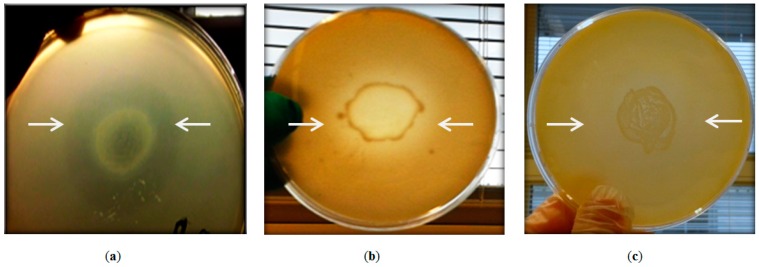
Antibacterial activity of *Geobacillus* sp. strain ZGt-1 against (**a**) *G.* *stearothermophilus* strain 10; (**b**) *B. subtilis*; and (**c**) *S. typhimurium* CCUG 31969. The arrows are pointing to the inhibition zone.

The ability of *Geobacillus* sp. ZGt-1 to inhibit the growth of the closely related *G. stearothermophilus* strain 10 was confirmed using the agar-deferred spot method (Figure 1). The antibacterial activity disappeared after treatment with proteinase K, indicating the proteinaceous nature of the secreted antibacterial agent. Testing of the antibacterial activity of ZGt-1 against mesophilic bacteria revealed inhibition zones in the case of *Bacillus subtilis* and the pathogenic *Salmonella typhimurium* CCUG 31969 (Figure 1), but not with *Escherichia coli* 1005, *Staphylococcus aureus* NCTC 83254, *Staphylococcus epidermidis*, and *Proteus vulgaris*.

The inhibition zone of ZGt-1 developed against the Gram-positive *B. subtilis* was more prominent than that against Gram-negative *S. typhimurium* (Figure 1), which may be ascribed to the different cell wall structures. In contrast to the Gram-negative bacteria, in which the cell wall peptidoglycan (PG) layer is protected by an outer membrane mainly composed of lipopolysaccharides (LPS), the PG in the Gram-positive bacteria is directly exposed to the external factors, including the cell wall lytic enzymes and antimicrobial peptides (AMPs) [13,14,15]. Although AMPs, but not cell wall lytic enzymes, can interact with the negatively charged outer membrane, some Gram-negative bacteria can develop species-specific mechanisms to eliminate the effect of the AMPs under certain environmental conditions [16,17,18]. Moreover, the variations in the structure of LPS, especially in Lipid A, among the different bacteria influence the AMP affinity and insertion into the outer membrane [16,17]. For example, there are differences in the polysaccharide chains and in Lipid A between *E. coli* and *S. typhimurium*; the latter has an additional fatty acid and different substituents of phosphate groups in Lipid A [19].

The differences in the susceptibility of the various Gram-positive bacteria (*B. subtilis* and *S. aureus*) to the antimicrobial action of ZGt-1 could be ascribed to the differences in the degree of crosslinking between the stem peptides (short peptide chains of 4–5 amino acids) linked to *N*-acetyl muramic acid residues in the PG [15]. In case of *B. subtilis*, 56%–63% of the stem peptides contribute to the cross-links, in contrast to *S. aureus*, in which up to 90% of the stem peptides are involved in the cross-linkages in the cell wall [15]. Moreover, the cross-links in *B. subtilis* as well as in *G. stearothermophilus* PG are direct (known as diaminopimelic acid (DAP)-direct), between d-alanine in the stem peptide of one glycan strand and the *meso*-diaminopimelic acid (*meso*-DAP) in another [20], while the cross-links in *S. aureus* form a pentaglycine bridge between l-lysine in the stem peptide of one glycan strand and d-alanine in another [21]. Even the charged polymers, teichoic- and teichuronic acids, which are covalently linked to the PG of Gram-positive bacteria, can influence the sensitivity of the cell wall to the lytic enzymes and AMPs, as the structure and amount of those polymers are strain- and species-specific [14,22,23]. Under certain conditions, *B. subtilis* produces atypical teichoic acid, which contains only the negatively charged phosphate groups that help in attracting the cationic AMPs to the cell surface [14]. However, some bacteria, like *S. aureus*, can alter the net surface negative charges of the bacterial surface or produce proteolytic enzymes capable of degrading AMPs as a resistance mechanism [14].

### 2.3. Production of the Antibacterial Substance(s) Using Immobilized Cells in Sequential Batch Mode with Cell Recycling

**Figure 2:**
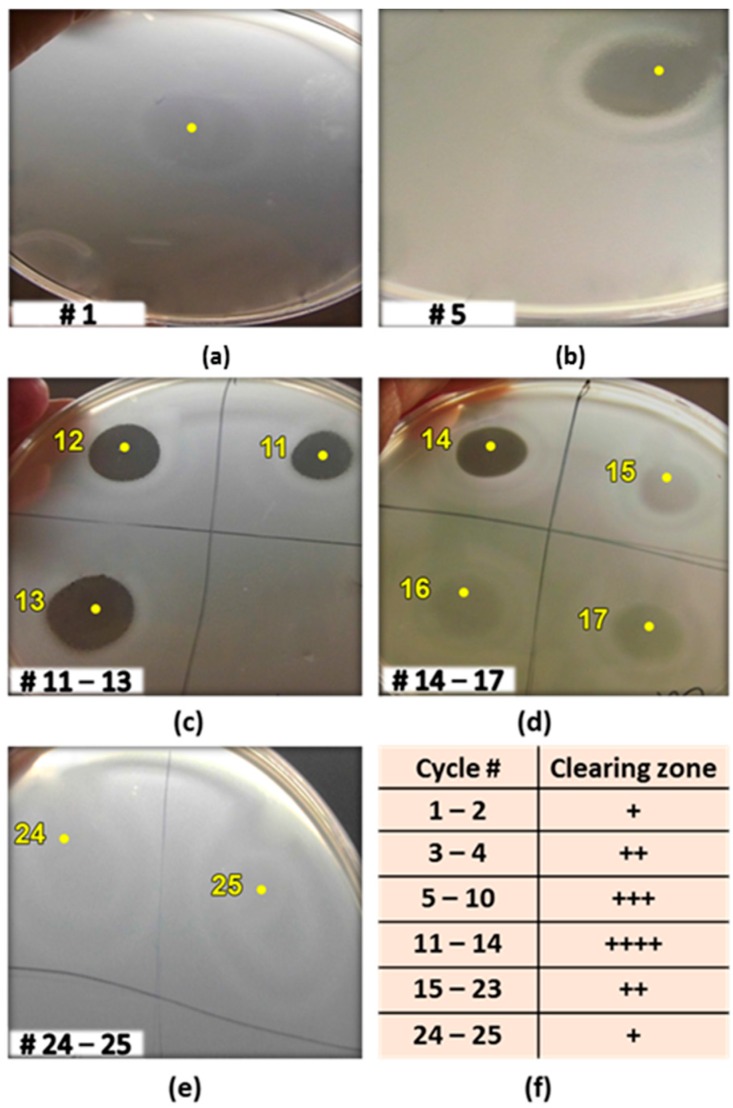
The antibacterial activity of the cell-free supernatant obtained by sequential batch cultivation of the immobilized cells of *Geobacillus* sp. ZGt-1 against *G. stearothermophilus* strain 10 at 60 °C, at the end of (**a**) cycle # 1; (**b**) cycle # 5; (**c**) cycles # 11, 12, and 13; (**d**) cycles # 14, 15, 16, and 17; (**e**) cycles # 24 and 25. The yellow dot denotes the site where the supernatant was spotted; and (**f**) Summary of the antibacterial activity over the 25 cycles, the number of + symbols representing increasing degree of antibacterial activity.

Although *Geobacillus* sp. ZGt-1 grown on solid MH agar medium exhibited good antibacterial activity against *G. stearothermophilus*, no antibacterial activity was observed on analysis of the culture supernatant when the isolate was grown overnight in a liquid medium (reaching an OD_620_ of 1.5). Similar observations have been reported previously with other strains [4,24]. Hence, it seems that the cell growth and production of antimicrobial agents is facilitated on the solid-state medium. To confirm this and to enable cultivation in larger scale for the production of antimicrobial molecule(s), the ZGt-1 cells were immobilized by entrapment in agar beads that were suspended in a liquid culture medium. This approach resulted in the appearance of antibacterial activity in the medium. The immobilized cells could be recovered and recycled for sequential batch cultivations in fresh medium with increasing antibacterial activity over consecutive cycles up to the 14th batch (Figure 2a–d). The bacterial growth during the cultivations was noted by the appearance of free cells in the medium. Entrapment inside the gel beads provides a protective environment for the cells that are more active at producing certain metabolites than the free cells and show increased tolerance to inhibitory compounds, which might otherwise limit the cell growth [25,26]. After the 14th cycle, however, the antimicrobial activity started to decrease and then disappeared almost completely during the 25th batch (Figure 2d,e). This decrease may be due to the nutritional limitations for the larger number of immobilized cells present and also the competition by the free cells that are not efficient in production of the antimicrobial metabolites [26], or due to difficulties with exporting the antimicrobial compound to the extracellular environment.

### 2.4. Antimicrobial Proteins Produced by Strain ZGt-1

**Scheme 1:**
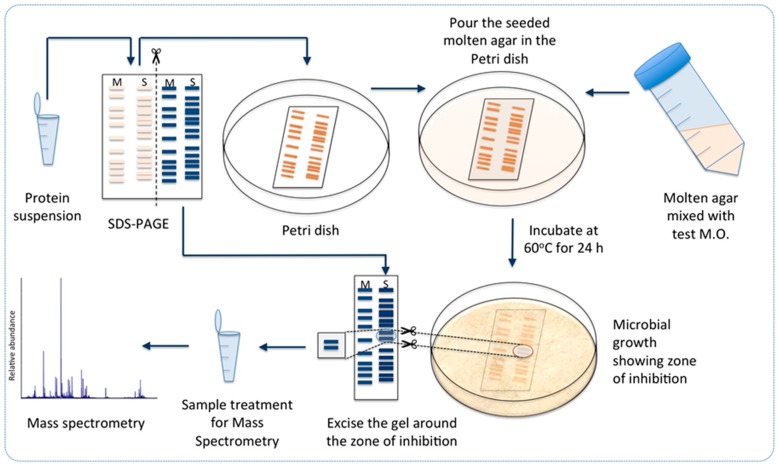
Workflow for identification of potential antimicrobial protein candidates in *Geobacillus* sp. ZGt-1: Desalted protein extract from *Geobacillus* sp. ZGt-1 was fractionated by SDS-PAGE, followed by detection of antibacterial activity on the gel against test microorganism (M.O.) and analysis of the active zone by mass spectrometry. S: sample, M: protein marker in the SDS-PAGE cartoons in the scheme. The experimental details are provided in the text.

**Figure 3:**
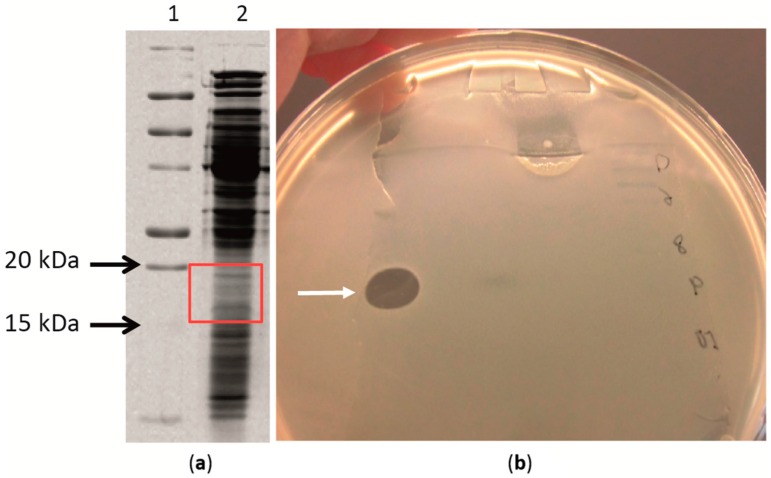
Analysis of the antibacterial activity of the desalted protein fraction isolated from the culture supernatant produced by *Geobacillus* sp. ZGt-1 at 60 °C. The test organism was *G. stearothermophilus* strain 10. After SDS-PAGE separation of the protein in duplicates, the gel was divided into two, one part stained with Coomassie Brilliant Blue R 250 (**a**) and the other used for antibacterial assay (**b**). (**a**) Image of the SDS-PAGE separated protein fraction. **Lane 1**: Precision Plus Protein All Blue standards; **Lane 2**: Desalted protein fraction; (**b**) Antibacterial activity of the desalted protein extract after separating it on SDS-PAGE separated protein; the gel strip was placed in a Petri dish and covered with soft agar layer seeded with strain 10 and incubated at 60 °C. The white arrow is pointing to the inhibition zone ascribed to the antibacterial activity of the protein fraction. The inhibition zone corresponded to 15–20 kDa.

Protease treatment gave us an indication of the antimicrobial activity to be associated to protein(s). Therefore, we proceeded with isolation of the proteins from the cell-free supernatant collected from the sequential batches with immobilized ZGt-1 cells by precipitation with ammonium sulphate. The protein precipitate obtained with 60% salt saturation was dialyzed against distilled water, and its antibacterial activity was confirmed using the spot-on-lawn method (Figure S1). The activity was found to be stable after heating at 70 °C for 45 min but was lost on heating to 80 °C for 10 min (Figure S2). The proteins in the sample were resolved on SDS-PAGE and the gel was subjected to the antibacterial activity test (Scheme 1). An area corresponding to 15–20 kDa molecular mass displayed the inhibition zone with *G. stearothermophilus* (Figure 3 and Figure S3).

Interestingly, the protein fraction was seen to be active even after being exposed to SDS under reducing conditions (with dithiothreitol; DTT), which was confirmed during at least eight separate SDS-PAGE-antibacterial activity assays performed using two different batches of the protein molecular weight standards. Higher abundance of SDS-resistant proteins has been reported in thermophilic microorganisms than that in the mesophilic ones [10]. Production of such proteins seems to be a defense strategy developed by prokaryotes, especially thermophiles, to protect some of their proteins against aggregation and premature degradation in order to save energy for thriving in extreme environments [10]. Moreover, the insensitivity of the antimicrobial activity to the reducing agent is possibly due to the lack of cysteine residues and hence the disulfide bridges, as is the case for several antimicrobial peptides [16].

### 2.5. Targeted Proteomics Analysis of the Antibacterial Protein-Containing Gel Samples

The gel area corresponding to 15–20 kDa, which displayed the inhibition zone against *G. stearothermophilus* strain 10, was analyzed for its protein content by liquid chromatography tandem mass spectrometry (LC-MS/MS). At the same time, the genome of *Geobacillus* sp. ZGt-1 was sequenced and annotated (GenBank accession no. LDPD00000000.1) [27], and used to construct a ZGt-1 strain-specific protein database. The collected MS/MS data were searched against the locally created ZGt-1 database to identify the proteins present in the excised gel samples. To be considered true protein identification, the following criteria had to be fulfilled: significant peptide sequences greater than or equal to 5, a total protein score greater than or equal to 200, and an individual peptide score greater than or equal to 20. Many proteins larger than 20 kDa were identified (Table S2), which could possibly be attributed to proteolysis. The high resolving power and sensitivity of the mass spectrometric analysis carried out in this study enables the detection of peptides of truncated or degraded proteins even if present in a small amount. Some proteolysis is indeed to be expected due to the action of bacterial proteases during the sequential batch cultivations and subsequent processing and storage of the extract at 4 °C until the time of analysis. Besides proteolysis, SDS-resistance could be another factor underlying the identification of proteins larger than 20 kDa. SDS-resistant proteins preserve their compact structure and display an apparent molecular weight in the gel that is smaller than their theoretical molecular weights [28,29].

Based on the antibacterial activity being limited to a molecular weight of 15–20 kDa, we decided to screen for proteins within the range of 10–30 kDa to take into account the “gel shifting” phenomenon, as noted above for the SDS-resistant proteins [28,29,30]. Table 1 lists the identified proteins, most of which are not known to display antibacterial activity. Only three uncharacterized/hypothetical proteins within 10–30 kDa were identified in two of the three excised gel samples, with 5–8 significant peptide sequences and a score of 292–517 (Table S3). We combined two approaches in a non-mutually exclusive way to predict the antibacterial activity of the uncharacterized proteins: calculation of the physiochemical properties of the proteins based on their amino acid sequences and comparing the inferred properties to those of known antimicrobial peptides/proteins, and *in silico* prediction of their antibacterial activity by employing the web-based antimicrobial peptides/proteins prediction algorithms, in an approach similar to that reported earlier [31,32] with some modifications.

### 2.6. Prediction of Antibacterial Potency Based on Physicochemical Properties

Table 2 summarizes the physicochemical properties depicted from analysis of the amino acid sequences of the three uncharacterized proteins. The proteins are in the molecular weight range of approximately 14–19 kDa, and a size range of 129–173 amino acid residues, which is in the range (10 to 100 s) for AMPs [33].

The antibacterial activity of many AMPs is brought about by lysis of the cells or membrane permeabilization due to electrostatic and/or hydrophobic interaction with the membrane of the target cell [34]. This interaction requires the AMP to have a net positive charge at neutral pH to help in initiating the interaction with the anionic surface of the bacterial cell, and to be rich in hydrophobic amino acid residues for insertion into the hydrophobic core of the bacterial cell membrane to destabilize the lipid bilayer and eventually kill the bacteria [32,34,35,36]. A minimum net charge of +2 is one of the unique features of cationic α-helical AMPs [37]. The three proteins have isoelectric points (pI) in the range of 7.72–8.80. Two of the proteins (ID 6_35, and 23_543) have a net charge of +2 at neutral pH, while all have a hydrophobic ratio of 36%–40% (Table 2).

The AMPs typically adopt an amphipathic conformation, i.e., the hydrophobic and polar residues are located on opposite sides. Linear amphipathic α-helical AMPs represent an important class of AMPs [34,38]. Analyzing the amino acid sequences of the three uncharacterized proteins using “The Antimicrobial Peptide Database (APD3)” showed that the three proteins have the potential to form amphipathic α-helices, where a certain number of hydrophobic amino acid residues group on the same side of the α-helix (Table 2), they are free of disulfide bridges, and have a linear amphipathic α-helical conformation. This is in agreement with the insensitivity of the antimicrobial effect to the reducing environment, as observed above (Section 2.4).

Grand average of hydropathy (GRAVY) index, a measure of the peptide/protein solubility, is within the range of −0.257 to −0.044 for the uncharacterized proteins (Table 2), in accordance with the predominant range of −1 to 0 for AMPs listed in the BIOPEP database as well as the predicted AMPs from milk proteins [32]. The Boman index values, reflecting the potential of a peptide/protein to interact with other proteins [38], for 6_35, 23_543, and 4_4 are 1.12, 1.57, and 1.19, respectively (Table 2), which also fit within the range of 1–2 for AMPs in the BIOPEP database [32].

In vitro stability of the AMPs reflects their bioavailability over a period of time [32]. The proteins 6_35, 23_543, and 4_4 have instability index values of 14.04, 17.61, and 36.7, respectively (Table 2), and are classified as stable in vitro, according to a criterion of instability index below 40 to be good for stability [39]. The tolerance to heat and SDS shown above could be a reflection of the stability of the proteins.

The Alpha index is a measure of the relative volume of the aliphatic amino acids side chains (alanine, leucine, isoleucine, and valine) [32,40], and has a positive correlation with thermostability [40]. The proteins 6_35, 23_543, and 4_4 scored 80.08, 110.33, and 96.76 on the aliphatic index, respectively (Table 2), again within the predominant range of 40–120 for AMPs in the BIOPEP database [32].

The potential of the AMPs to form aggregates at their site of interaction with the bacterial cell membranes is a necessary step for their mechanism of action [35]. AGGRESCAN software [41] predicted that there are three, six, and six putative aggregation “hot spots” within the sequences of the proteins, 6_35, 23_543, and 4_4, respectively (Table 2). The Na^4^vSS values were 5.3, −6.6, and −2.4 for 6_35, 23_543, and 4_4, respectively (Table 2). Previously reported AMPs have Na^4^vSS values within the range of −40 to 60 [36,42].

### 2.7. *In Silico* Prediction of the Antibacterial Potency

The bioinformatics tools used for predicting the antibacterial potency of the uncharacterized proteins based on the amino acid sequences were the sequence-based prediction tools available on “The Collection of Anti-Microbial Peptides (CAMP_R3_)” [43], the antimicrobial peptide calculator and predictor tool available on the “APD3” [44], and the “AMPA” application [45].

The CAMP_R3_ prediction tool uses machine learning algorithms to predict the antimicrobial activity of peptides/proteins including the novel ones. CAMP_R3_ uses four different prediction models (Support Vector Machines (SVM), Random Forests (RF), Artificial Neural Network (ANN), and Discriminant Analysis (DA)). SVM, RF, and DA predict the antimicrobial activity and state the AMP probability, while ANN makes a qualitative description of the peptide/protein as either an AMP (for “antimicrobial peptide/protein”) or NAMP (for “non-antimicrobial peptide/protein”). The accuracy of the prediction results for the models is within the range of 87%–93% [32,46].

APD3 predicts the potential of an amino acid sequence (up to 200 residues) to have an antimicrobial activity by analyzing each amino acid residue and comparing the physicochemical properties of the sequence with those of the natural AMPs already deposited in the APD3 database [32,44,47]. AMPA uses a sliding window algorithm that calculates an “antimicrobial index” for individual amino acid residues and estimates the tendency of the amino acid to be found within an AMP sequence. By doing so, AMPA identifies the antimicrobial domain within the protein/peptide and hence predicts the overall antimicrobial activity [45]. The algorithms indicated that the three uncharacterized proteins have antimicrobial potential to different extents (Table 2).

Combining the prediction results inferred from the sequence-derived physicochemical properties with the results of the six prediction algorithms used (the four algorithms of CAMP_R3_, the APD3 algorithm, and the AMPA algorithm), we can conclude that protein 23_543 is the most likely antibacterial protein candidate (Table 2). In the case of the protein 6_35, not all six prediction algorithms confirmed its potential as an antimicrobial protein. Protein 4_4 does not fulfill all the physicochemical properties of AMPs because its pI is 7.72 and its positive net charge is +1 [37]. Moreover, not all six prediction algorithms confirmed the potential of protein 4_4 as an antimicrobial protein. However, the probability of the antimicrobial potential of these two proteins cannot be ignored since most of the sequence-derived properties match with the features known about reported AMPs, and four out of the six algorithms predicted them to display antimicrobial activity (Table 2).

### 2.8. Identified Protein Sequences Matching Parts of Antimicrobial Enzymes

As described above (Section 2.5), many proteins larger than 20 kDa were identified by LC-MS/MS analysis. Among these are two enzymes (26_23 and 2_3 in Table S2) already reported as antibacterial; *N*-acetylmuramoyl-l-alanine amidase (referred to as amidase; EC.3.5.1.28), and serine-type d-alanyl-d-alanine carboxypeptidase (referred to as dd-carboxypeptidase; EC: 3.4.16.4). The predicted amino acid sequences of amidase and dd-carboxypeptidase in strain ZGt-1 give a theoretical molecular weight of approximately 87 and 47 kDa, respectively, after cleavage of the enzyme’s signal peptide. These enzymes are known to catalyze the lysis of bacterial cells by hydrolyzing the covalent bonds in the peptidoglycan layer of the bacterial cell wall [13,48]. Amidase cleaves the amide bond between the glycan and the peptide chain [13], while dd-carboxypeptidase cleaves the terminal d-alanyl-d-alanine bond in the stem peptides of the peptidoglycan layer, resulting in the removal of the terminal d-alanine [49]. The lytic enzymes of bacterial origin are known to have a role in bacterial cell growth and division [13,48], and also act as antimicrobials by attacking the cell wall of competing bacteria [13].

Matching of the MS/MS-detected significant peptide sequences of the ZGt-1 amidase (Table S3) to the enzyme domains that we previously identified using InterProScan analysis [50] of the predicted amino acid sequence, showed alignment with segments in the catalytic domain at N-terminus (data not shown). Similar analysis of ZGt-1 dd-carboxypeptidase domains showed a match between the MS/MS-detected significant peptide sequences of the enzyme (Table S3) with N-terminus segments (the catalytic domain), C-terminus segments (supposed to act as the enzyme’s binding domain as indicated by the InterProScan tool), and parts of the region in between the two domains (data not shown). However, whether the detected partial sequences of the two enzymes would be responsible for the antibacterial activity and the clearance zone in the 15–20 kDa region is not clear.

## 3. Materials and Methods

### 3.1. Materials

R2A broth, containing per liter: 0.5 g meat peptone, 0.5 g casamino acids, 0.5 g yeast extract, 0.5 g dextrose, 0.5 g soluble starch, 0.3 g dipotassium hydrogen phosphate, 0.05 g magnesium sulphate, and 0.3 g sodium pyruvate (pH 7.2 ± 0.2), was purchased from Lab M (Heywood, UK), while Bacto agar was from Difco, BD (Detroit, MI, USA). MH agar, having a composition per liter of 2 g meat infusion, 17.5 g casein hydrolysate, 1.5 g starch and 17.0 g agar (pH 7.3 ± 0.2), was procured from Merck (Darmstadt, Germany). MH broth, having per liter 17.5 g acid hydrolysate of casein, 3.0 g beef extract, 1.5 g starch (pH 7.3 ± 0.1), was purchased from BBL, BD (Sparks, MD, USA). Proteinase K (≥30 Units/mg) was obtained from Sigma-Aldrich (St. Louis, MO, USA).

### 3.2. Isolation of Bacteria from Zara Hot Spring in Jordan, Strain Maintenance and Cultivation Conditions

Water samples were collected from the shores of Zara hot spring (with water temperature of 46 °C and pH 7) in Jordan (32 N 36 E) from 15 to 25 cm depth under the water surface level using sterile pipettes and dispensed into sterile falcon tubes. Within an hour after sampling, 500 μL of the samples were spread on the surface of solid R2A agar (1.7% *w*/*v*) plates, which were incubated aerobically at 60 °C for two days. The resulting bacterial colonies were isolated and further purified by streaking onto new R2A agar plates, followed by overnight incubation at 60 °C. The pure colonies were then suspended in 16% *v*/*v* glycerol to prepare a stock culture suspension and stored at −80 °C.

Cultivation of the bacterial isolates was done by streaking one loopful of the culture stock onto the surface of R2A agar plates for isolation of DNA, or MH agar plates for production of antibacterial molecules. The plates were incubated overnight at 60 °C. Subsequently, 1–2 colonies were chosen, re-streaked on the respective media, and incubated under similar conditions. The resulting colonies were used either for DNA extraction, testing the antibacterial activity (agar-deferred spot method), or as an inoculum for production experiments.

### 3.3. Identification of the Bacterial Isolates by 16S rRNA Sequencing

DNA was extracted from the pure cultures obtained above using ZR Fungal/Bacterial DNA MiniPrep (Zymo Research, Orange, CA, USA). 16S rRNA genes were amplified by PCR using universal primers, 10–30 F (5′-GAGTTTGATCCTGGCTCA-3′) and 1500 R (5′-AGAAAGGAGGTGATCCAGCC-3′) (Eurofins, Ebersberg, Germany). The reaction mixture (50 μL) contained 1× Phusion^®^ GC buffer with 1.5 mM MgCl_2_, 200 μM of each dNTP, 0.4 μM of each primer, 3% dimethyl sulfoxide, 1 U Phusion^®^ Hot Start II DNA polymerase (Finnzymes, Thermo Fisher Scientific, Helsinki, Finland), 5 ng of the purified DNA, and the final volume was adjusted with nuclease free water. The PCR reaction was carried out in Whatman Biometra T-gradient thermocycler (Biometra GmbH, Göttingen, Germany) under the following conditions: initial denaturation at 98 °C for 30 s, followed by 25 cycles of denaturation at 98 °C for 10 s, primer annealing at 61 °C for 30 s, extension at 72 °C for 23 s, and final extension at 72 °C for 600 s. The PCR products were visualized on 1.2% (*w*/*v*) agarose gel stained with GelRed™ 3× (Biotium Inc., Fremont, CA, USA), purified and sequenced in the forward and reverse directions by ABI sequencing reaction (GATC Biotech, Konstanz, Germany). The 16S rRNA sequences obtained were compared against sequences available in GenBank by applying the BLASTn 2.3.1+ [51] using the Megablast option on the RefSeq_RNA database (NCBI Transcript Reference Sequences).

### 3.4. Detection of Antibacterial Activity of *Geobacillus* sp. ZGt-1

#### 3.4.1. Agar-Deferred Spot Method

For determination of antibacterial activity by this method, several bacteria were used as test strains including a closely related thermophilic strain *Geobacillus stearothermophilus* (strain 10), isolated from the same environmental niche, and mesophilic *Bacillus subtilis* TMB94 and pathogenic bacterial strains, *E. coli* 1005, *S. aureus* NCTC 83254, *S. epidermidis* TMB96, *S. typhimurium* CCUG 31969, and *P. vulgaris* TMB02. The bacterial cultures were streaked onto MH agar plates that were incubated overnight at 60 and 37 °C, respectively. Colonies of the respective test strains were then suspended in sterile saline solution (0.85% *w*/*v* NaCl) and used to seed soft MH agar (0.5×) pre-warmed at 55 °C in case of *G. stearothermophilus*, or at 45 °C for the mesophiles, such that the final OD_550_ was ~0.125 (equivalent to 0.5 McFarland turbidity standard).

One or two colonies of *Geobacillus* ZGt-1 were spot-inoculated onto the center of MH agar plates that were incubated aerobically at 60 °C for 24 h, and subsequently overlaid with soft agar seeded with the test strain, and incubated for 15–18 h at 60 °C (*G. stearothermophilus* strain 10) or for 22–24 h at 37 °C (mesophiles) to allow the test strains to grow. The presence/absence of the inhibition zone around the spot of strain ZGt-1 was detected.

#### 3.4.2. Spot-on-Lawn Method

Ten milliliters of soft agar seeded with the test strain (*G. stearothermophilus* strain 10) were poured into Petri dishes and allowed to solidify. Fifty microliters of the cell-free culture supernatant or desalted protein fraction (after ammonium sulphate precipitation) from *Geobacillus* sp. ZGt-1 were spotted on the agar surface and incubated at 60 °C for 15–18 h. The presence/absence of the inhibition zone around the spot was detected.

### 3.5. Sensitivity of the Antibacterial Substance(s) to Proteolysis by Proteinase K

Sensitivity of the antibacterial activity towards proteinase K was tested as described earlier [52] with slight modifications. *Geobacillus* sp. ZGt-1 cells were spot-inoculated at the centre of MH agar plates and incubated overnight at 60 °C. Thereafter 3 μL of proteinase K solution (2 mg/mL) was spotted on one side of the ZGt-1 grown spot, while the other side was not treated, and the plate was incubated for 2 h at 37 °C. Finally, the plate was overlaid with 5 mL of soft MH agar seeded with *G. stearothermophilus* strain 10 using the agar-deferred spot method described in Section 3.4.1, and then incubated overnight at 60 °C.

### 3.6. Batch Production of the Antibacterial Substance(s) in Shake Flasks by Free Cells of *Geobacillus* sp. ZGt-1

Ten milliliters of MH broth in a 50-mL sterile Falcon tube were inoculated with 1–2 colonies of *Geobacillus* sp. ZGt-1 and incubated in an orbital shaker incubator (IKA KS 4000 ic, Staufen, Germany) at 60 °C and 240 rpm to reach a final OD_620_ of 0.468. Two milliliters of the resulting culture were transferred to 200 mL of the broth in a 500-mL baffled Erlenmeyer flask that was incubated under similar conditions to reach the final OD_620_ of 1.5. Finally, the culture was centrifuged at 10,000× *g* for 20–30 min at room temperature and the supernatant was tested for antibacterial activity.

### 3.7. Sequential Batch Production of the Antibacterial Substance(s) in Shake Flasks Using Immobilized Cells of *Geobacillus* sp. ZGt-1

Cells were cultivated and harvested as described above (Section 3.6). The cell pellet was then re-suspended in 10 mL of fresh sterile MH broth, and mixed with 20 mL of 3% (*w*/*v*) sterile molten agar solution pre-warmed at 60 °C. The mixture was dropped into a sterile cold solution of oil: saline solution (1:2) using a syringe needle to form beads with an average diameter of 5 mm. The beads were washed thoroughly with sterile saline solution and distilled water to remove the oil and then transferred to the 500-mL cultivation flask containing 200 mL of fresh sterile MH broth. The bead suspension was incubated at 60 °C, 240 rpm for 22–25 h, after which the culture broth was separated from the beads by carefully pouring it into sterile centrifuge bottles. The broth was then centrifuged at 10,000× *g* for 20–30 min at room temperature and the supernatant was filtered using 0.2 µm syringe filters, and saved.

The beads were transferred back to the cultivation flask with 100 mL of fresh sterile MH broth, and another cultivation cycle was carried out. The beads were recycled 25 times in the same manner (except during cycles 12 and 17, where 10 µM FeCl_3_ was supplemented to the medium to be used for another study), and the cell-free supernatants were tested for antibacterial activity by the spot-on-lawn approach.

### 3.8. Towards Identification of the Antibacterial Protein Candidates

The cell-free supernatant collected from sequential cultivation batches in Section 3.7, except for batches 12 and 17, were pooled together, and 1400 mL of the solution were treated with 60% (*w*/*v*) ammonium sulphate and allowed to stand with mild stirring at 4 °C for about 15 h. The precipitate was separated by centrifugation at 10,000× *g*, 4 °C for 40 min and re-suspended in 10 mL distilled water, yielding 140 times enrichment, and dialyzed using a dialysis membrane (3.5 kDa cutoff) (Spectra Pro) against 5 liters of distilled water that was freshly replaced several times a day for three days to remove the ammonium sulphate. To ensure removal of ammonium sulphate, which in itself may have an inhibitory effect, an aliquot of fresh MH culture medium was also subjected to a similar treatment and used as a negative control. The dialyzed samples were tested for their antibacterial activity by the spot-on-lawn approach.

### 3.9. Sensitivity of the Antibacterial Protein(s) to Heat

Thermostability of the protein fraction isolated above was tested by heating 500 μL aliquots of the desalted sample at 70 °C for 10, 15, 30, and 45 min, or at 80 °C for 10 and 15 min, respectively, in a heating block and then placed on ice for 45–60 min. The samples were then centrifuged for 10 min at 13,000× *g* at room temperature to separate the denatured proteins, and the supernatant was tested for antibacterial activity by applying the spot-on-lawn technique.

### 3.10. Sensitivity of the Antibacterial Protein(s) to SDS and Fractionation by SDS-PAGE

Twenty microliters of the protein sample was mixed with 5 μL of loading buffer (50 mM Tris-HCl pH 6.8, 100 mM DTT, 2% (*w*/*v*) SDS, 0.1% bromophenol blue, 10% (*v*/*v*) glycerol). The mixture was incubated at room temperature for 30–60 min, and then loaded on polyacrylamide gel (with 15% *w*/*v* acrylamide) in duplicates. After electrophoresis, the gel was cut into two halves, each with a lane of the sample and molecular weight standard (Precision Plus Protein All Blue standards, Biorad, Hercules, CA, USA); one half was stained with Coomassie Brilliant Blue R 250 while the other was tested for antibacterial activity by the method described earlier [53]. For the latter, the gel was fixed immediately in a solution of 20% isopropanol and 10% acetic acid for 2 h, washed in distilled water for 6 h, placed in a sterile Petri dish and overlaid with 15 mL of soft MH agar (0.5×) seeded with the test strain (*G. stearothermophilus* strain 10), and incubated at 60 °C for 12 h for development of the inhibition zone (Scheme 1).

### 3.11. Targeted Proteomics Analysis of the Antibacterially Active Protein Fraction Using Mass Spectrometry

The gel area that showed the inhibition zone against *G. stearothermophilus* strain 10 was excised and divided into three gel samples. Each gel sample was cut into 1 × 1 mm pieces and placed in high-recovery Eppendorf tubes, washed twice in 75 µL of 50 mM ammonium bicarbonate/50% ethanol mixture to remove the Coomassie Brilliant Blue stain, dehydrated in 75 µL 100% ethanol, and then subjected to reduction (10 µL of 10 mM DTT, incubated at 37 °C for 30 min) and alkylation (10 µL of 55 mM iodoacetamide, incubated in dark for 30 min), with a second dehydration step in between. The gel pieces were washed and dehydrated once again as described above, and then 10 µL of digestion buffer (50 mM ammonium bicarbonate with 12 ng/µL sequencing-grade modified trypsin (Promega, Madison, WI, USA)) were added. After incubation on ice for 1 h, 15 µL of 50 mM ammonium bicarbonate was added and the samples were digested overnight at 37 °C. Subsequently, the digestion solution was withdrawn and the peptides were further extracted by adding 15 µL of 1% trifluoroacetic acid (TFA). After incubation for 2 h, this extract was collected and pooled with the overnight digestion solution.

The tryptic peptide mixtures were further fractionated by reversed phase nano-LC prior to mass spectrometric analyses, using an LTQ-Orbitrap Velos Pro mass spectrometer (Thermo Fisher Scientific) equipped with a nanoEasy spray ion source (Proxeon Biosystems, Odense, Denmark). The chromatographic separation was performed at 40 °C on a 15 cm (75 μm i.d.) EASY-Spray column packed with 3 μm resin (Proxeon Biosystems). The nanoHPLC intelligent flow control gradient was 5%–20% solvent B (0.1% *v*/*v* FA, 100% *v*/*v* acetonitrile in water) in solvent A (0.1% *v*/*v* FA in water) for 60 min, and then 20%–40% solvent B for 30 min, followed by an increase to 90% for 5 min. A flow rate of 300 nL/min was used throughout the whole gradient. The MS scan (usually 350–2000 *m*/*z*) was recorded in the Orbitrap mass analyzer set at a resolution of 60,000 at 400 *m*/*z*, 1 × 10^6^ automatic gain control target, and 500 ms maximum ion injection time. The MS was followed by data-dependent collision-induced dissociation MS/MS scans on the eight or 10 most intense multiply charged ions in the LTQ at 15,000 signal threshold, 30,000 automatic gain control target, 300 ms maximum ion injection time, 2.5 *m*/*z* isolation width, 10 ms activation time at 35 normalized collision energy, and dynamic exclusion enabled for 30 or 60 s with a repeat count of 1.

The general mass spectrometric conditions were as follows: spray voltage, 2.0 kV; no sheath or auxiliary gas flow; S-lens 60%; ion transfer tube temperature, 275 °C. The Mascot Server software v. 2.4 (http://www.matrixscience.com) [54] was used for protein identification and Mascot Distiller was used to generate high-quality, de-isotoped peak lists from the raw data files. In parallel, gene prediction was carried out on the ZGt-1 genome [27] using prodigal (v2_60.linux) [55] and identified coding sequences of genes were then translated into proteins. A database of ZGt-1 proteins was constructed, and the MS/MS-identified peptides were searched against the ZGt-1 protein database (parameter settings used: trypsin-specific digestion, digestion with one missed cleavage site, peptide mass tolerance 10 ppm, fragment ion mass tolerance ±0.15 Da, carbamidomethylation set as fixed modification) and the proteins that accounted for those peptides were interpreted. In another step, protein identification was also obtained by matching the mass spectrometric data by Mascot searches directly in UniProt, with protein identification based on sequence homology to proteins in other bacterial species. The proteins that had significant peptide sequences more than or equal to 5, with ion score ≥20, and a total protein score ≥200, were considered as true matches.

### 3.12. UniProt Database Search for Identification of the MS/MS-Identified Proteins Using BLASTp

The proteins from strain ZGt-1 interpreted from the MS/MS-detected peptides (Section 3.11) were identified with the aid of BLASTp version 2.2.30+ [56] using UniProt [57] as database. The e-value threshold was set to 1 × 10^−10^ and the top hit was used for annotation of protein description.

### 3.13. *In Silico* Analysis of the Proteins Identified as Possible Antimicrobials

#### 3.13.1. Calculating the Physicochemical Properties of the Uncharacterized Proteins

The physicochemical properties of the uncharacterized proteins (6_35, 23_543, and 4_4) were calculated using computational tools freely available on the internet. The number of amino acid residues, molecular mass, pI, net charge, instability index, aliphatic index, and GRAVY index were calculated using the ProtParam tool on the ExPasy webserver [39]. The amphipathic helix formation, total hydrophobic ratio, number of hydrophobic residues located on the same side of the helix, Boman index, and confirmation of the net charge were calculated using the antimicrobial peptide calculator and predictor available on the Antimicrobial Peptide Database (APD3) [44].

Aggregation of the proteins in vivo was predicted using AGGRESCAN [41], where putative aggregation hot spots and normalized average of aggregation propensity (Na^4^vSS) of the input protein were calculated. “Hot spot” is a protein aggregation region with a minimum of 5 continuous amino acid residues with an average aggregation propensity higher than a certain threshold calculated by the AGGRESCAN algorithm and none of the residues is a proline, which is an aggregation breaker [41]. The Na^4^vSS value is the sum of aggregation propensities for the amino acids of the input protein divided by the number of amino acid residues in the protein sequence and multiplied by 100 [41].

#### 3.13.2. Prediction of Antimicrobial Activity of the Uncharacterized Proteins

The antimicrobial activity of the uncharacterized proteins (6_35, 23_543, and 4_4) was predicted using the sequence-based prediction tools freely available on the web, the Collection of Anti-Microbial Peptides (CAMP_R3_), where we employed the “AMP prediction” tool and used the four available prediction models (Support Vector Machines (SVM), Random Forests (RF), Artificial Neural Network (ANN), and Discriminant Analysis (DA)) [43]. The prediction results were retrieved as either a score evaluating the probability of the protein to be antimicrobially active (in case of using SVM, RF, and DA), or as a description of the protein either as AMP (for antimicrobial peptide/protein), or NAMP (for non-antimicrobial peptide/protein) (in case of using ANN). We also used another prediction tool, the antimicrobial peptide calculator and predictor available on the APD3 [44]. The third tool used was the AMPA server [45], where the default settings were used. Afterwards, we compared the output results retrieved from the different prediction tools to assess the antimicrobial potential of the respective protein.

#### 3.13.3. Domain Architecture Analysis of Antimicrobial Enzyme Sequences

The functional domain prediction of the amidase and dd-carboxypeptidase enzyme sequences was performed using the InterProScan sequence search tool [50].

## 4. Conclusions

The thermophilic bacterial isolate, *Geobacillus* sp. ZGt-1, seems to be a promising source of several antimicrobial molecules including proteins, which was confirmed by the availability of its genome sequence [27]. This study showed the potential of the isolate in inhibiting the growth of the dairy- and food-spoiling thermophilic bacteria and some other mesophiles including pathogens. Importantly, the potential of combining the immobilized cell technology with cell-recycling for the production of antimicrobial proteins was demonstrated that could be applicable even for other environmental isolates.

Combining the proteomics data with the genome sequence information revealed the presence of as yet uncharacterized proteins with antimicrobial potential, and also the presence of antimicrobial enzymes. Further work will deal with empirical confirmation of the antimicrobial activity of the individual protein candidates suggested in this study (typed in bold in Table S2). The putative candidates will be cloned and expressed in order to investigate the scope of their antibacterial activity and the possibility of their synergistic action.

## Figures and Tables

**Table 1:** Proteins (10–30 kDa) identified by mass spectrometry.

Query ID	MS Score	Mw (kDa) ^1^	Homologous Protein Name ^2^	UniProt ID
2_80	253	17.118	2-C-methyl-d-erythritol 2,4-cyclodiphosphate synthase	G8N0X9
23_188	469	16.072	6,7-Dimethyl-8-ribityllumazine synthase	L8A0J9
28_41	488	20.475	ATP synthase subunit b	G8MZV8
186_1_184_1	219	22.862	Capsid protein	A0A0K9I0I6
190_1_188_1	243	15.718	Capsid protein	A0A0K9I0I6
23_393	258	23.554	Deoxyribose-phosphate aldolase	A0A063YQK6
4_30	219	17.987	DinB family protein	U2WSJ5
26_1	403	19.041	Flagellin	L8A2E4
23_543	517	16.846	Hypothetical conserved protein	Q5KWM5
23_84	241	19.549	Menaquinol-cytochrome c reductase iron-sulfur subunit	S7U299
6_3	293	17.044	N5-carboxyaminoimidazole ribonucleotide	Q5L3D8
23_103	610	16.741	Nucleoside diphosphate kinase	G8MZM9
13_48	231	20.528	Peptide deformylase	Q5L138
23_492	803	21.126	Peroxiredoxin	Q5KWS6
23_704	866	18.266	Probable thiol peroxidase	Q5KW64
28_65	267	23.049	Probable transaldolase	L8A4Q9
23_775	357	16.545	Starvation-induced protein controlled by σ-B	Q5KVZ0
25_145	314	27.457	Triose phosphate isomerase	A0A063YNF6
4_4	432	19.025	Uncharacterized protein	Q5L3L9
6_35	406	13.884	Uncharacterized protein	Q5L3A8
18_68	208	27.963	Uroporphyrin-III C-methyltransferase	Q5KZ09
21_9	315	19.432	YceI family protein/uncharacterized protein	G8MXK2

^1^ Theoretical molecular weight based on the amino acid sequence; ^2^ Top hits of the UniProt BLASTp searches, all e-values were significant and much less than or equal to 0.

**Table 2:** Prediction of the antimicrobial potential of the uncharacterized proteins based on their physicochemical properties and algorithm models.

Physicochemical Properties				
Property		Protein Query ID		
		6_35	23_543	4_4
Length		129	153	173
Molecular weight (kDa)		13.8927	16.8564	18.979.1
Net charge		+2	+2	+1
pI		8.80	8.61	7.72
Instability index		14.04	17.61	36.7
Aliphatic index		80.08	110.33	96.76
GRAVY index		−0.044	−0.257	−0.253
Boman index (kcal/mol)		1.12	1.57	1.19
Na^4^vSS		5.3	−6.6	−2.4
Number of aggregation hot spot regions		3	6	6
Total hydrophobic ratio		40%	39%	36%
Potential of forming amphipathic helix		Yes	Yes	Yes
Number of hydrophobic residues on the same side		≥38	≥42	≥31
**Algorithm Models**				
CAMP_R3_ Models	SVM	1.000 ^1^	1.000 ^1^	1.000 ^1^
	RF	0.987 ^1^	0.9575 ^1^	0.991 ^1^
	DA	1.000 ^1^	1.000 ^1^	1.000 ^1^
	ANN	NAMP ^2^	AMP ^3^	NAMP ^2^
APD3		AMP ^3^	AMP ^3^	AMP ^3^
AMPA		NAMP ^2^	AMP ^3^	NAMP ^2^
			(0.86) ^1^	
**Summary of the Fulfilled Antimicrobial Potential Parameters Inferred from the Physicochemical Properties and Prediction Algorithms**				
Physicochemical properties		All	All	Majority
Prediction algorithms		Majority	All	Majority

^1^ Probability of being an antimicrobial peptide/protein; ^2^ Non-antimicrobial peptide/protein; ^3^ Antimicrobial peptide/protein.

## Supplementary Materials

Supplementary materials can be found at www.mdpi.com/1422-0067/17/8/1363/s1.

## Abbreviations

Amidase	*N*-acetylmuramoyl-l-alanine amidase
AMP	Antimicrobial peptide/protein
ANN	Artificial neural network
APD3	Antimicrobial peptide database
CAMP_R3_	The collection of anti-microbial peptides
DA	Discriminant analysis
dd-carboxypeptidase	Serine-type d-alanyl-d-alanine carboxypeptidase
DTT	Dithiothreitol
GRAVY	Grand average of hydropathicity
LPS	Lipopolysaccharide
LC-MS/MS	Liquid chromatography tandem mass spectrometry
*meso*-DAP	*Meso*-diaminopimelic acid
MH	Mueller Hinton culture medium
Na^4^vSS	Normalized average of aggregation propensity
NAMP	Non-antimicrobial peptide/protein
PG	Peptidoglycan
pI	Isoelectric point
RF	Random forests
SVM	Support vector machines
