# Supplementary material for: Antimicrobial Protein Candidates from the Thermophilic Geobacillus sp. Strain ZGt-1: Production, Proteomics, and Bioinformatics Analysis
Source: Int J Mol Sci. 2016 Aug 19;17(8):1363. doi: 10.3390/ijms17081363 (PMC5000758; doi:10.3390/ijms17081363)
Supplement: Supplementary file 1 [file ijms-17-01363-s001.pdf]

Supplementary Materials: Antimicrobial Protein Candidates from the Thermophilic *Geobacillus* sp. Strain ZGt-1: Production, Proteomics, and Bioinformatics Analysis

Rawana N. Alkhalili, Katja Bernfur, Tarek Dishisha, Gashaw Mamo, Jenny Schelin, Björn Canbäck, Cecilia Emanuelsson and Rajni Hatti-Kaul

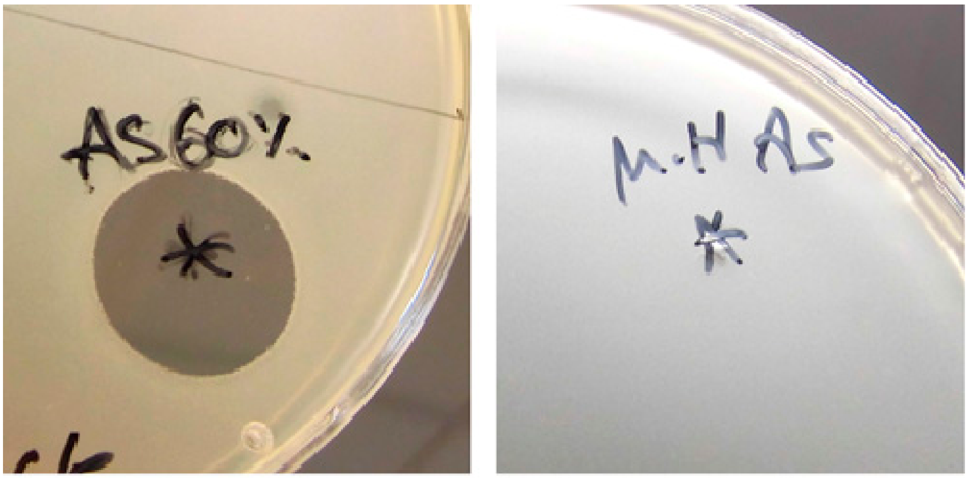

(a) (b)

Figure S1. Antibacterial activity of the (**a**) concentrated and desalted protein fraction from the culture supernatant obtained after batch cultivation of immobilized *Geobacillus* sp. ZGt-1 cells; and (**b**) concentrated and desalted Mueller Hinton broth (negative control), tested by the spot-on-lawn method. Fifty microliters of the samples were spotted over a layer of soft Mueller Hinton agar seeded with *G. stearothermophilus* strain 10 in two separate Petri dishes. The antibacterial activity was observed only in the case of (**a**).

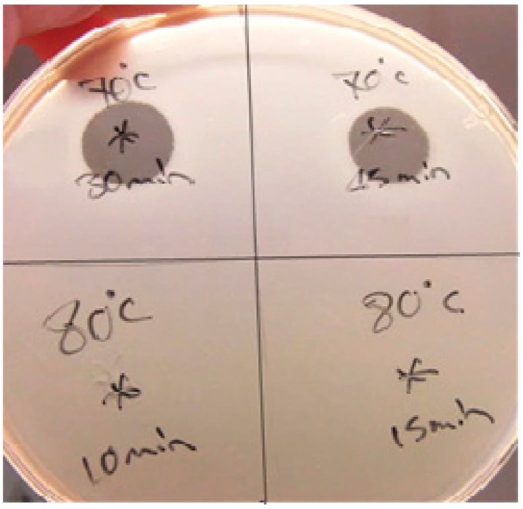

Figure S2. Antibacterial activity of the concentrated and desalted protein fraction after heating at 70 °C for 30 and 45 min, and at 80 °C for 10 and 15 min. Fifty microliters of the samples were spotted over a layer of soft Mueller Hinton agar seeded with *G. stearothermophilus* strain 10.

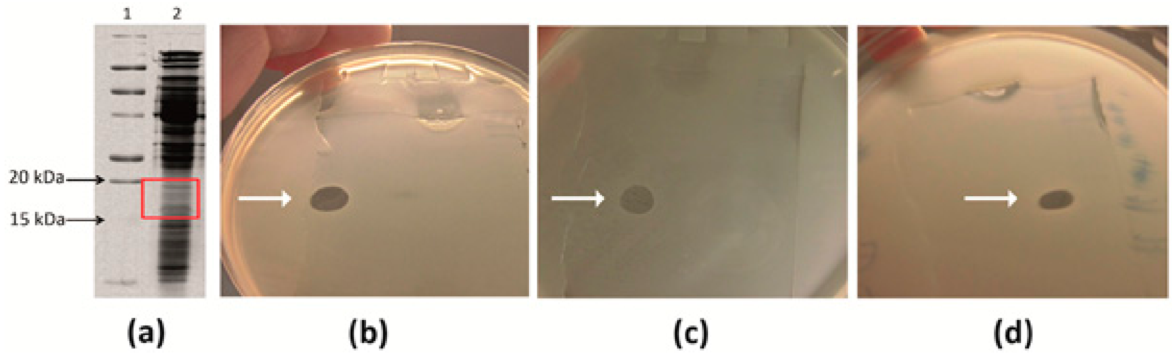

Figure S3. Analysis of the antibacterial activity of the desalted protein fraction isolated from the culture supernatant produced by *Geobacillus* sp. ZGt-1 at 60 °C. The test organism was *G. stearothermophilus* strain 10. (**a**) Gel image of the SDS-PAGE separated protein fraction. **Lane 1**: Precision Plus Protein All Blue standards; **Lane 2**: Desalted protein fraction; (**b**–**d**) Plates representing the results of three independent assays; in each assay, the treated gel strip was placed in a Petri dish and covered with soft agar layer seeded with strain 10 and incubated at 60 °C. White arrows point to the inhibition zones produced due to the antibacterial activity of the protein fraction. The inhibition zone corresponded to the 15–20 kDa range in all the replicates.

Table S1. BLASTn (2.3.1+) analysis results of the 16S rRNA gene sequence. [1]

Isolate Designation	Best Matched Identity	Matched Identity [2] (%)	Query Cover (%)	Identities	Length of the Sequenced Fragment of the Gene
Strain ZGt-1 (GenBank accession no. KT026965)	*Geobacillus kaustophilus* strain BGSC 90A1	100.0	100	1454/1454 residues	1454
	Geobacillus thermoleovorans, strain LEH-1	99.9		1453/1454 residues	
Strain 10 (GenBank accession no. KU933578)	*Geobacillus stearothermophilus* strains (R-35646; NBRC 12550; BGSC 9A20; DSM 22; IFO12550)	99.6	99	1405/1411 residues	1415

[1] BLASTn analysis was last checked on 23 May 2016; [2] Matched identity (%) had an e-value of 0.0. BLASTn analysis of 16S rRNA gene of strain ZGt-1 indicated that the strain belongs to the genus *Geobacillus*. At the time of writing this paper, a matched identity of 99.9%–100% to the16S rRNA genes of *G. kaustophilus* and *G. thermoleovorans* strains was found (Table S1). BLASTn analysis of 16S rRNA gene of strain 10 indicated that the strain is best aligned with *G. stearothermophilus* (Table S1).

Table S2. Proteins identified by mass spectrometry of the SDS polyacrylamide gel, corresponding to 15–20 kDa region, which showed the inhibition zone against *G. stearothermophilus* strain 10.

Query ID	MS Score	Mw (kDa)	Homologous Protein Name	UniProt ID	Taxon ID *,a	Gel Sample *,b
23_788	506	30.298	1,4-Dihydroxy-2-naphthoyl-CoA synthase	Q5KVX8	2	1,3
2_80	253	17.118	2-C-methyl-D-erythritol 2,4-cyclodiphosphate synthase	G8N0X9	4	2
13_266	248	31.174	4-Hydroxy-tetrahydrodipicolinate synthase	G8N1Y8	4	1,2,3
26_51	615	85.418	5′-Nucleotidase domain protein	G8MXU7	4	1,2,3
23_188	469	16.072	6,7-Dimethyl-8-ribityllumazine synthase	L8A0J9	8	1,2
18_137	410	54.566	6-Phospho-beta-glucosidase	V6VF75	9	1,2,3
8_169	264	46.557	ABC transporter substrate-binding protein	T0PXY1	5	1
23_721	217	65.139	Acetyl-CoA synthetase	Q5KW45	2	1
13_336	1440	99.980	Aconitate hydratase	G8N2L5	4	1,2,3
25_107	206	52.678	Adenosylmethionine-8-amino-7-oxononanoate aminotransferase	T0NWM1	5	2
6_5	410	50.017	Adenylosuccinate lyase	Q5L3D6	2	3
26_67	430	51.713	Aldehyde dehydrogenase	A0A063YKS3	7	1,2,3
23_754	669	59.790	Alkaline metalloprotease	A0A0K0Q2H9	1	1,2,3
3_2	677	32.513	Arginase	V6V9A1	9	1
28_41	488	20.475	ATP synthase subunit b	G8MZV8	4	3
6_13	493	55.023	Bifunctional purine biosynthesis protein	G8N332	4	1,2,3
186_1_184_1	219	22.862	Capsid protein	A0A0K9I0I6	3	2
190_1_188_1	243	15.718	Capsid protein	A0A0K9I0I6	3	1
17_1	648	78.072	Catalase-peroxidase	Q9S5Q0	3	1,2,3
23_398	412	65.848	Chaperone protein DnaK	G8N2T3	4	1,3
171_3_169_3	491	33.261	CRISPR-associated protein DevR	A0A0K9HTL1	3	1,2,3
13_50	363	35.476	Dehydrogenase E1 component, beta subunit (Lipoamide)	Q5L136	2	1,2,3
23_393	258	23.554	Deoxyribose-phosphate aldolase	A0A063YQK6	6	2
13_52	971	49.544	Dihydrolipoyl dehydrogenase	Q5L134	2	1,2,3
23_270	639	50.445	Dihydrolipoyl dehydrogenase	Q5KXC2	2	1,2,3
13_51	281	47.152	Dihydrolipoyllysine-residue acetyltransferase component of pyruvate dehydrogenase complex	G8MZH5	4	1
4_30	219	17.987	DinB family protein	U2WSJ5	2	1
2_100	371	77.174	Elongation factor G	A0A0D8BQU2	2	2,3
25_143	1080	46.573	Enolase	G8MWZ8	4	1,2,3
8_126	295	36.350	Ferrochelatase	U2YAF2	2	1,2,3
26_1	403	19.041	Flagellin	L8A2E4	8	1,2,3
28_66	247	31.073	Fructose-bisphosphate aldolase	Q5KUG5	2	1
23_372	324	40.768	GTP cyclohydrolase 1 type 2 homolog	Q5KX23	2	1,3
**23_543**	**517**	**16.846**	**Hypothetical conserved protein**	**Q5KWM5**	**2**	**1,3**
23_653	309	46.656	Isocitrate dehydrogenase [NADP]	Q5KWB6	2	1,2,3
13_127	396	10.5192	Isoleucine--tRNA ligase	T0NQC8	5	2,3
23_272	828	40.739	Leucine dehydrogenase	G5EBC7	2	1,2,3
2_69	321	57.275	Lysine--tRNA ligase	T0PWW3	5	2
8_167	222	60.848	Maltogenic amylase	O69007	11	2
23_84	241	19.549	Menaquinol-cytochrome c reductase iron-sulfur subunit	S7U299	10	2
**26_23**	**223**	**89.226**	***N*-acetylmuramoyl-L-alanine amidase**	**G8MXR3**	**4**	**3**
6_3	293	17044	N^5-carboxyaminoimidazole ribonucleotide	Q5L3D8	2	1,2
23_103	610	16741	Nucleoside diphosphate kinase	G8MZM9	4	1
12_77	385	66009	Oligoendopeptidase, M3	G8MYR3	4	1,3
4_16	559	44509	Ornithine aminotransferase	Q5L3K7	2	1,2,3
13_48	231	20528	Peptide deformylase	Q5L138	2	3
23_492	803	21126	Peroxiredoxin	Q5KWS6	2	3
23_764	454	58571	Phosphoenolpyruvate carboxykinase [ATP]	Q5KW01	2	1,2,3
8_32	266	65607	Phosphomannomutase	Q5L2H5	2	2,3
18_76	308	44778	Precorrin-3B methylase CobJ fused to precorrin-6B methylase 2 CobL	G8N796	4	1
23_271	236	40290	Probable butyrate kinase	Q5KXC1	2	1
23_704	866	18266	Probable thiol peroxidase	Q5KW64	2	3
28_65	267	23049	Probable transaldolase	L8A4Q9	8	3
**2_3**	**223**	**51225**	**Serine-type D-Ala-D-Ala carboxypeptidase**	**Q5L3Y3**	**2**	**1**
23_775	357	16545	Starvation-induced protein controlled by sigma-B	Q5KVZ0	2	1
26_54	1898	192778	surface layer glycoprotein SgsE	U2WRU3	2	2,3

Table S2. *Cont.*

Query ID	MS Score	Mw (kDa)	Homologous Protein Name	UniProt ID	Taxon ID *,a	Gel Sample *,b
23_726	462	36590	Transcriptional regulator involved in in carbon catabolite control	A0A098L0H0	4	3
13_321	486	72034	Transketolase	T0Q680	5	2,3
25_145	314	27457	Triosephosphate isomerase	A0A063YNF6	7	1
**4_4**	**432**	**19025**	**Uncharacterized protein**	**Q5L3L9**	**2**	**1,3**
**6_35**	**406**	**13884**	**Uncharacterized protein**	**Q5L3A8**	**2**	**1,2**
93_1	1360	47857	Uncharacterized protein	T0PW66	5	2,3
18_68	208	27963	Uroporphyrin-III C-methyltransferase	Q5KZ09	2	1
21_9	315	19432	YceI family protein/uncharacterized protein	G8MXK2	4	3
23_747	234	51673	Xaa-His dipeptidase	A0A098KZ03	4	2,3
23_304	456	38937	Xaa-Pro dipeptidase	Q5KX90	2	3

* Protein identification was obtained by matching the mass spectrometric data by Mascot searches either directly in UniProt, by sequence homology to proteins in other bacterial species, or in a two-step procedure matching the data first to the local database created based on the genome sequence of *Geobacillus* sp. strain ZGt-1 [27] followed by the identification by UniProt BLASTp-searches. UniProt BLASTp top hit proteins identified in other bacterial species with significant e-values (4.00×10^{-157} to 0.0) are presented in columns **Homologous protein name** and **Taxon ID** *,a respectively; *,a The numbers in the **Taxon ID** *,a column correspond to the following organisms: 1: *B. subtilis*, 2: *G. kaustophilus,* 3: *G. stearothermophilus,* 4: *G. thermoleovorans*, 5: *Geobacillus* sp. A8, 6: *Geobacillus* sp. CAMR12739, 7: *Geobacillus* sp. CAMR5420, 8: *Geobacillus* sp. GHH01, 9: *Geobacillus* sp. MAS1, 10: *Geobacillus* sp. WSUCF1, 11: *Thermus* sp; *,b The gel area that showed the inhibition zone against *G. stearothermophilus* was excised and divided into three gel samples, 1, 2, and 3, with each sample analyzed separately. Proteins identified fulfill the following requirements: significant peptide ≥5, protein score ≥200, and peptide ion scores ≥20.

Table S3. Amino acid sequences of detected peptides in the proteins identified as possible antimicrobials.

Protein Query	Unique Peptides	Mw (Da) (expt.) [2]	Ion Charge	Ion Score
**Uncharacterized protein (6_35)**				
Gel sample 1 [406 (6)] [1]	R.FQGDTELASANK.A	1279.6015	+2	104
	R.FQGDTELASANKATNSK.S	1780.8523	+3	24
	K.SKVENSVVGADYK.A	1394.7000	+2	88
	K.VENSVVGADYK.A	1179.5742	+2	35
	K.VSPTALNVTDNDTR.Y	1501.7295	+2	107
	K.VSPTALNVTDNDTRYFTVDQSAVGK.K	2697.3149	+3	110
Gel sample 2 [352 (7)] [1]	R.FQGDTELASANK.A	1279.6013	+2	102
	R.FQGDTELASANKATNSK.S	1780.8536	+3	50
	K.SKVENSVVGADYK.A	1394.7005	+2	67
	K.VSPTALNVTDNDTR.Y	1501.7287	+2	99
	K.VSPTALNVTDNDTRYFTVDQSAVGK.K	2697.3143	+3	55
	R.YFTVDQSAVGK.K	1213.5948	+2	29
	R.YFTVDQSAVGKK.V	1341.6898	+3	22
**Hypothetical protein (23_543)**				
Gel sample 1 [292 (6)] [1]	R.LAIAADNNTK.T	1029.5427	+2	59
	R.LAIAADNNTK.T	1029.5427	+2	43
	R.LAIAADNNTKTSYTLK.Q	1722.9063	+3	34
	K.QLYDDGYLENIPK.S	1566.7493	+2	88
	K.VTLVDDDGEFVYIDGSK.D	1870.8750	+2	86
	K.VTLVDDDGEFVYIDGSKDVNELTR.K	2698.2871	+3	48
Gel sample 3 [517 (9)] [1]	R.LAIAADNNTK.T	1029.5426	+2	74
	R.LAIAADNNTK.T	1029.5427	+2	53
	K.QLYDDGYLENIPK.S	1566.7516	+2	79
	K.QLYDDGYLENIPKSPGK.H	1935.9538	+3	65
	K.ETDEKNNVTGILYK.V	1622.8100	+2	87
	K.ETDEKNNVTGILYK.V	1622.8102	+3	20
	K.NNVTGILYK.V	1020.5575	+2	60
	K.VTLVDDDGEFVYIDGSK.D	1870.8775	+2	115
	K.VTLVDDDGEFVYIDGSKDVNELTR.K	2698.2894	+3	67
**Uncharacterized protein (4_4)**				
Gel sample 1 [432 (7)] [1]	K.TIGDLVVLSGNSNLPK.G	1625.8924	+3	40
	K.TIGDLVVLSGNSNLPK.G	1625.8942	+2	91
	K.GAVVQIVMK.E	943.5480	+2	48
	K.QVLEEKVNVGEDGSYSWSAK.R	2224.0552	+3	97
	K.VNVGEDGSYSWSAK.R	1497.6652	+2	78
	K.DSSGRVEYQTDGQTYVGIK.M	2101.9830	+3	59
	R.VEYQTDGQTYVGIK.M	1599.7712	+2	97
Gel sample 3 [390 (7)] [1]	K.TIGDLVVLSGNSNLPK.G	1625.8932	+3	60
	K.TIGDLVVLSGNSNLPK.G	1625.8941	+2	69
	K.GAVVQIVMK.E	943.5497	+2	66
	K.QVLEEKVNVGEDGSYSWSAK.R	2224.0564	+3	49
	K.VNVGEDGSYSWSAK.R	1497.6660	+2	96
	K.DSSGRVEYQTDGQTYVGIK.M	2101.9856	+3	52
	R.VEYQTDGQTYVGIK.M	1599.7731	+2	77
**Amidase (protein 26_23)**				
Gel sample 3 [223 (6)] [1]	R.VSYSLLPGNR.L	1104.5902	+2	44
	R.HYSDRLALK.I	1101.5917	+3	38
	K.ILETGLK.G	772.4680	+2	21
	K.IIIDAGHGAHDTGAIGPGGTR.E	1985.0004	+3	83
	K.IIIDAGHGAHDTGAIGPGGTR.E	1985.0032	+4	35
	R.STDIFLELSER.T	1308.6529	+2	61
**DD-Carboxypeptidase (protein 2_3)**				
Gel sample 1 [223 (5)] [1]	R.ADAAILVDAQTGR.I	1299.6732	+2	80
	K.NIDTVLGIASMTK.M	1361.7171	+2	53
	R.ALSNVPLRK.D	996.6055	+2	46
	K.FVNATGLSNK.D	1049.5472	+2	60
	R.VPLVTTTEVEK.A	1214.6722	+2	34

[1] Significant protein score (number of significant peptides)] in the respective gel sample; [2] Mw (expt.): Theoretical molecular weight expected from the amino acids sequence.
